# National-scale geodatabase of catchment characteristics in the Philippines for river management applications

**DOI:** 10.1371/journal.pone.0281933

**Published:** 2023-03-08

**Authors:** Richard J. Boothroyd, Richard D. Williams, Trevor B. Hoey, Craig MacDonell, Pamela L. M. Tolentino, Laura Quick, Esmael L. Guardian, Juan C. M. O. Reyes, Cathrine J. Sabillo, John E. G. Perez, Carlos P. C. David

**Affiliations:** 1 School of Geographical & Earth Sciences, University of Glasgow, Glasgow, United Kingdom; 2 School of Geography, Earth & Environmental Sciences, University of Birmingham, Birmingham, United Kingdom; 3 Department of Civil & Environmental Engineering, Brunel University London, Uxbridge, United Kingdom; 4 National Institute of Geological Sciences, University of the Philippines, Diliman, Philippines; 5 Department of Geography & Regional Research, University of Vienna, Vienna, Austria; University of Bucharest, ROMANIA

## Abstract

Quantitative descriptions of stream network and river catchment characteristics provide valuable context for enabling geomorphologically-informed sustainable river management. For countries where high-quality topographic data are available, there are opportunities to enable open access availability of baseline products from systematic assessment of morphometric and topographic characteristics. In this study, we present a national-scale assessment of fundamental topographic characteristics of Philippine river systems. We applied a consistent workflow using TopoToolbox V2 to delineate stream networks and river catchments using a nationwide digital elevation model (DEM) acquired in 2013 and generated through airborne Interferometric Synthetic Aperture Radar (IfSAR). We assessed morphometric and topographic characteristics for 128 medium- to large-sized catchments (catchment area > 250 km^2^) and organised the results in a national-scale geodatabase. The dataset realises the potential of topographic data as part of river management applications, by enabling variations in hydromorphology to be characterised and contextualised. The dataset is used to reveal the diversity of stream networks and river catchments in the Philippines. Catchments have a continuum of shapes (Gravelius compactness coefficient ranges from 1.05 to 3.29) with drainage densities that range from 0.65 to 1.23 km/km^2^. Average catchment slope ranges from 3.1 to 28.1° and average stream slope varies by more than an order of magnitude from 0.004 to 0.107 m/m. Inter-catchment analyses show the distinctive topographic signatures of adjacent river catchments; examples from NW Luzon highlight topographic similarity between catchments whereas examples from Panay Island shown marked topographic differences. These contrasts underline the importance of using place-based analyses for sustainable river management applications. By designing an interactive ArcGIS web-application to display the national-scale geodatabase, we improve data accessibility and enable users to freely access, explore and download the data (https://glasgow-uni.maps.arcgis.com/apps/webappviewer/index.html?id=a88b9ca0919f4400881eab4a26370cee). The national-scale geodatabase provides a baseline understanding of fundamental topographic characteristics in support of varied geomorphological, hydrological and geohazard susceptibility applications.

## 1. Introduction

Topography is an important driver of hydrological, geomorphological and ecological processes in global river systems. As such, quantitative descriptions of stream network and river catchment characteristics are essential for enabling geomorphically-informed sustainable river management. At the catchment-scale, the configuration of stream networks and physiographic properties of river catchments influences water flow and sediment flux across landscapes [1]. Morphometric analyses provide a quantitative description of stream networks and river catchments through the assessment of linear (e.g., stream length), areal (e.g., drainage density) and relief (e.g., slope) characteristics; calculated from elevation data of the Earth’s surface typically in the form of digital elevation models (DEMs). Morphometric and topographic characteristics have been used to interpret processes relevant to a variety of hydrological and geomorphological applications. Reflecting the hydrologic response of a catchment [2] they provide hydromorphological information on flood-conditioning factors [3]. Indicating the internal dynamics of sediment transport and storage they have been used to predict catchment-scale sediment fluxes [4]. Relevant to longer-term landscape evolution they indicate the influence of climate regimes on the nature and intensity of erosional processes [5]. Importantly, morphometric and topographic characteristics provide a means of observing and assessing the extent to which variation occurs within and between catchments [6].

Large-scale DEMs with near global coverage (e.g., Shuttle Radar Topography Mission, SRTM) have been used to generate hydrographic information (e.g., HydroSHEDS [7], HydroRIVERS [8]) and assess globally distributed morphometric and topographic characteristics (e.g., [9–11]). Higher quality topographic datasets are becoming increasingly available with nationwide (e.g., 1 m resolution LIDAR in the United Kingdom [12] and Taiwan [13]), regional (e.g., 2 m resolution ArcticDEM [14]) and global coverages (e.g., 12 m resolution TanDEM-X [15] and 30 m resolution FABDEM [16]). Improvements in topographic data quality have coincided with the release of efficient processing tools designed to support the semi-automated analyses of river systems [17], including those for morphometric and topographic analyses (e.g., [18–20]). Higher quality topographic datasets have contributed to improved positional accuracy in stream network mapping applications (e.g., [21]) and semi-automated processing tools enable morphometric and topographic characteristics to be assessed with greater accuracy over larger extents than ever before (e.g., [22]).

The Philippines is one of the most vulnerable countries in Southeast Asia to climate change impacts [23] and consistently ranks as one of the most globally affected countries by extreme weather [24, 25]. Climate change is impacting the magnitude and frequency of extreme flood-generating storms [26, 27] and a high proportion of the population are exposed to hazards arising from fluvial flooding and erosion [28]. Challenges associated with water resource management (e.g., water security [29]) and hydrometeorological hazards (e.g., floods [30] and rainfall-triggered landslides [31]) have necessitated the acquisition of high-quality topographic datasets, primarily for resource mapping and predictive hazard modelling purposes. A nationwide topographic dataset with 5 m spatial resolution and 1 m root-mean-square error vertical accuracy was acquired in 2013 using airborne Interferometric Synthetic Aperture Radar (IfSAR) from the National Mapping and Resource Information Authority (NAMRIA) [32]. Higher resolution topographic data with a 1 m spatial resolution were acquired between 2012 and 2016 for major floodplains using airborne Light Detection and Ranging (LIDAR) from the Disaster Risk and Exposure Assessment for Mitigation (DREAM) and Phil-LiDAR 1 programs (https://dream.upd.edu.ph/) [33].

High-quality topographic data have been used to realise a step-change in hydrometeorological hazard information in the Philippines; underpinning nationwide landslide susceptibility assessments and detailed flood hazard reports for more than 300 river catchments [34]. Detailed maps for landslide, flood and storm surge hazards are used by Local Government Units (LGUs) for disaster preparedness planning and communicated to wider audiences through a web-based disaster Geographic Information System (Web-GIS) from the Nationwide Operational Assessment of Hazards (NOAH) program (https://noah.up.edu.ph/). The high-quality topographic data have also been used for resource mapping applications as part of the Phil-LiDAR 2 Program [33, 35]. Alongside additional remote sensing products (e.g., satellite imagery), the topographic data have been used to map stream networks, river catchments, irrigation networks and inland wetlands through the Development of the Philippine Hydrologic Dataset for Watersheds from LiDAR Surveys (PHD project) [36, 37]. The hydrological dataset contains information on 770 principal river basins (catchment area > 40 km^2^) but attribute information is limited to a small number of simple descriptors (e.g., catchment area and length of stream network lines).

Despite the availability of high-quality topographic data with a nationwide coverage, a systematic national-scale assessment of fundamental topographic characteristics of stream networks and river catchments has yet to be undertaken. Achieving such an analysis has the potential to provide a baseline dataset for geomorphologically-informed sustainable river management that is similarly significant to the transformation of flood risk management by airborne LiDAR. For water resource planning, the Philippines is divided into 12 water resources regions and 18 major river basins; designated by the National Water Resources Council [38]. Although existing river basin management plans contain information on catchment topography (e.g., qualitative and quantitative descriptions of terrain), the plans are only available for some of the larger river basins (catchment area > 3000 km^2^). For smaller catchments, inconsistent methods have been applied to derive topographic information for a variety of research purposes (e.g., geochemical mapping of the Santo Tomas River [39], fluvial morphology assessments in the Bislak catchment [40], streamflow predictions in the Abuan catchment [41], land use change impacts on hydrology in the Calumpang catchment [42]). Inter-catchment comparisons have previously been undertaken for small numbers of catchments (e.g., catchments with similar hydrological regimes [43]) or across a limited geographical extent (e.g., individual islands [44]). To date, it has not been possible to contextualise the topographic characteristics of stream network and river catchments at the national-scale, because: (1) fundamental topographic characteristics of small- to medium-sized catchments (catchment area < 3000 km^2^) are largely unquantified; (2) existing topographic analyses have limited interoperability (i.e., characteristics were calculated using inconsistent methods); and, (3) derived datasets are infrequently made accessible to end-users.

In this study, we systematically assess fundamental topographic characteristics of stream networks and river catchments in the Philippines. Using high-quality topographic data, we apply a consistent workflow to calculate topographic characteristics for 128 medium- to large-sized river catchments (catchment area > 250 km^2^). We provide a national-scale assessment of selected topographic characteristics before making detailed inter-catchment comparisons to illustrate similarities and differences between adjacent catchments. Our findings reveal the topographic diversity of stream networks and river catchments; individual catchments have distinctive topographic signatures and we observe substantial variation between catchments. This finding underpins the need to use the dataset for geomorphologically-informed sustainable river management applications, some of which we highlight. To improve data accessibility, we host our national-scale geodatabase in an interactive ArcGIS web-application that enables users to freely view, explore and download the data.

## 2. Materials and methods

**Fig 1:**
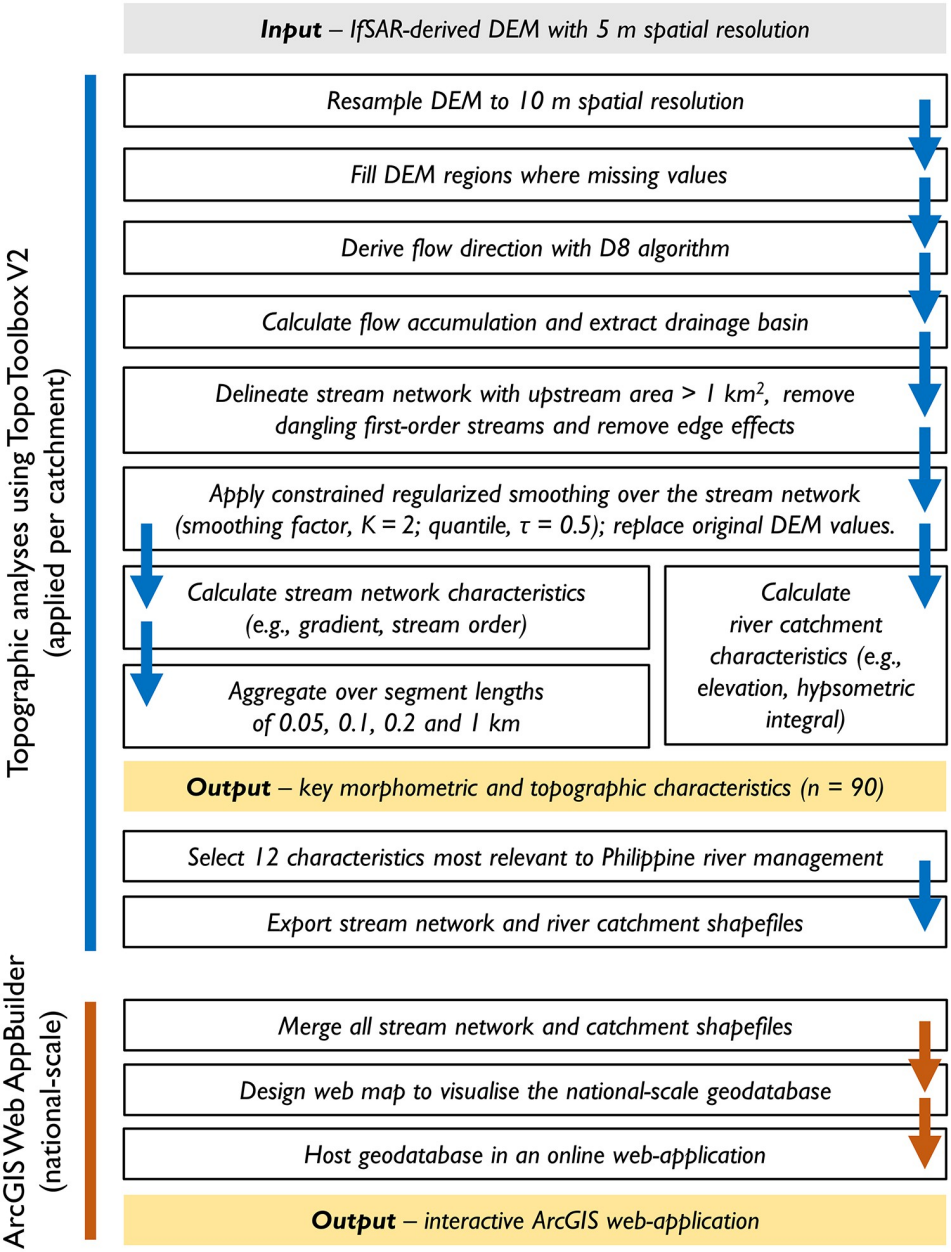
Workflow for calculating fundamental topographic characteristics of stream networks and river catchments from a DEM and displaying results in an interactive ArcGIS web-application.

In this section we summarise the workflow used to calculate fundamental topographic characteristics and display the results in an interactive ArcGIS web-application. The workflow is summarised in Fig 1, indicating the main processing steps and resultant products.

### 2.1. Topographic analyses

We used a nationwide IfSAR DEM acquired in 2013 for our analysis; it has a 5 m spatial resolution and covers approximately 300,000 km^2^ of the Philippine landmass [34]. Although higher resolution LIDAR data is available for the floodplains of more than 300 river catchments, the spatial coverage of the LIDAR data is incomplete (i.e., topographic data are not available for all parts of every catchment). Furthermore, by using the IfSAR DEM we maintained a consistent vertical accuracy at the national-scale (1 m root-mean-square error [34]). Due to computational constraints associated with processing the topographic data, we bilinearly resampled the 5 m IfSAR DEM to a 10 m spatial resolution before completing the analyses.

We used TopoToolbox V2 to delineate stream networks and river catchments using standard flow-routing algorithms [18] with the D8 algorithm used to derive flow direction. Having calculated flow accumulation and extracted individual drainage basins, we used an upstream area threshold value (1 km^2^) to delineate alluvial channels to be included within the stream network [45]. The upstream area threshold value marked the transition from debris flow-dominated channels to alluvial channels. The position of the DEM-derived stream networks were in good visual agreement with the position of channels in Google Earth imagery and within close proximity to the stream networks mapped through the PHD program [36, 37]. DEMs typically contain artefacts and errors that propagate into topographic analyses [46], meaning that derived attributes such as stream slope vary over short distances [47]. We ensured downstream decreasing elevations and provided a hydrologically correct DEM by applying constrained regularized smoothing to the stream network to minimise DEM artefacts and errors (smoothing factor, K = 2; quantile, τ = 0.5; [48]). Several attributes were extracted at all points along the stream network, including the elevation, drainage area, stream slope, stream order and distance from outlet. Because stream network points were densely spaced (0.01 km spacing; equal to DEM resolution), we aggregated points over river segment lengths of 0.05, 0.1, 0.2 and 1 km to generalize any local fluctuations in attribute values (Table 1). The segments split the stream network into homogeneous reaches of a set length; these were evenly distributed between confluences, confluences and outlets, and confluences and channel heads. The approach ensured that a consistent delineation method was applied to all stream networks and river catchments.

**Table 1:** Stream network point aggregation over varying segment lengths (0.05, 0.1, 0.2 and 1 km). Note that 1 km segment lengths were included in the ArcGIS web-application.

Stream network attribute	Units	Aggregation method	Description
Elevation	m	Mean	Average elevation over the segment length
Catchment area	km^2^	Median	Median average drainage area over the segment length
Stream slope	m/m	Mean	Average stream gradient of the channel over the segment length
Stream order	-	Maximum	Maximum Strahler stream order over the segment length
Distance from outlet	km	Mean	Average distance to the catchment outlet over the segment length

Following stream network and river catchment delineation, we calculated a set of morphometric and topographic characteristics (*n* = 91). This included linear, areal and relief characteristics routinely calculated as part of morphometric analyses, in addition to slope characteristics extracted along the stream network. An extensive literature exists on the selection of morphometric characteristics for hydrological and geomorphological applications (e.g., [49–51]); we selected characteristics most widely applicable to river management applications in the Philippines.

We systematically applied our workflow to 128 catchments (catchment area > 250 km^2^). The area threshold was selected to limit analyses to medium- to large-sized catchments. The geographical coverage of the selected catchments included the island groups of Luzon, Catanduanes, Mindoro, Samar, Leyte, Panay, Palawan, Negros, Bohol and Mindanao. Underlain by heterogeneous geologies, many of the catchments are influenced by active tectonic structures [52]. The catchments are distributed across the full range of Coronas climate types [27], likely resulting in varied hydrological regimes. The diverse geologic, tectonic and climatic settings of the catchments likely generate spatial differences in the hydromorphological characteristics of stream networks and river catchments.

### 2.2. ArcGIS web-application

**Fig 2:**
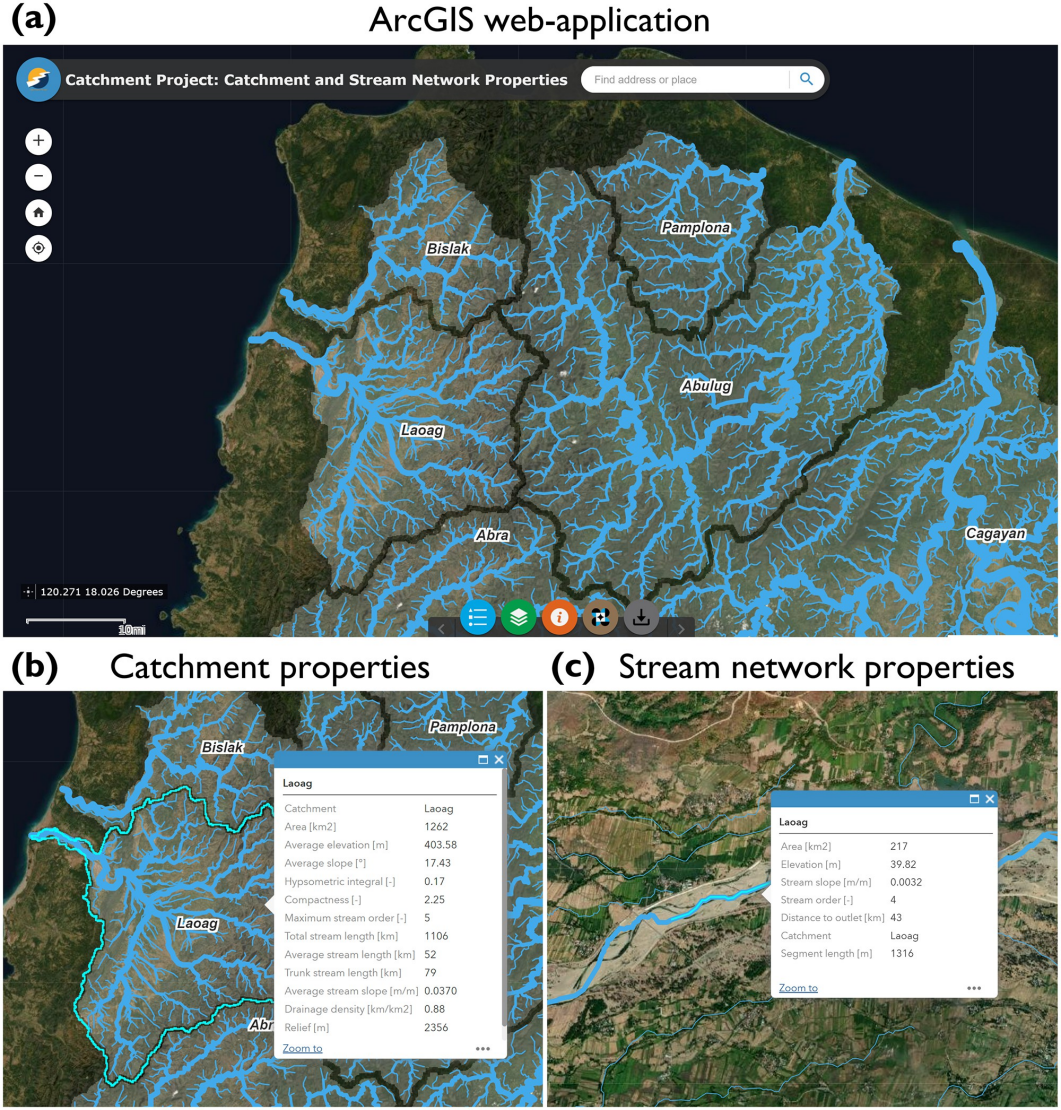
(a) Examples of Philippine river catchments and stream networks displayed in the ArcGIS web-application (basemap from Esri). Attributes of the Laoag catchment (Luzon Island) showing: (b) catchment properties and (c) stream network properties.

We designed an ArcGIS web-application to display a simplified version of the stream network and river catchment geodatabase. We used the ArcGIS Web AppBuilder to build the ArcGIS web-application (https://www.esri.com/en-us/arcgis/products/arcgis-web-appbuilder/overview). The ArcGIS web-application can be accessed from any device, improving accessibility to the data. Moreover, the ArcGIS web-application does not require users to be registered or have software-specific expertise; anyone can freely view, explore and interact with the stream network and river catchment geodatabase. The ArcGIS web-application displays stream network and river catchment layers overlain on an aerial image basemap (Fig 2a). The data layers are interactive; clicking on the river catchment layer opens a pop-up window containing quantitative information on catchment characteristics (Fig 2b) and clicking on the river network layer opens a pop-up window containing attribute information for the nearest river segment(s) (Fig 2c). The stream network layer is displayed using a symbology weighted by the Strahler stream order to display relative differences in river size. Descriptions of each of the morphometric and topographic characteristics are included within the web-application and links are provided to download the underlying data in open data formats. Additional functionality is provided whereby users can filter the layers to display features that match user-defined expressions (e.g., define an alternative catchment area threshold).

Simplifications were made to reduce the volume of data displayed in the web-application. The dense point spacing of the stream network provided excessive detail and caused data handling and drawing issues when displayed in the web-application. To reduce the volume of data we aggregated stream network points over 1 km segments (Table 1). The post-processed stream network for the 128 catchments contained 109,050 segments with an average length of 1.30 km (standard deviation = 0.37 km). The stream network for the web-application retained the positional accuracy of the original stream network, but reduced the number of data points by a factor of 100. A mask was applied to omit parts of the stream network that were routed through large water bodies (see Section 4.3 for a discussion of this limitation). We selected 12 of the most widely used morphometric and topographic characteristics for river management applications to display in the simplified geodatabase (Table 2). The simplifications ensured that the user-experience is consistent for anyone accessing the web-application.

**Table 2:** Selected attributes included in the ArcGIS web-application.

Catchment attribute	Units	Description
Area	km^2^	Drainage area of the catchment
Average elevation	m	Mean elevation of the catchment
Relief	m	Difference between the minimum and maximum elevation in the catchment
Average catchment slope	°	Mean slope in the catchment
Hypsometric integral	-	Scalar value to describe the area distribution at different elevations
Maximum stream order	-	Maximum Strahler stream order in the catchment
Total stream length	km	Combined length of streams in the catchment (1 km^2^ drainage area threshold)
Average stream length	km	Mean length of streams in the catchment (1 km^2^ drainage area threshold)
Trunk stream length	km	Length of the longest stream in the catchment (1 km^2^ drainage area threshold)
Average stream slope	m/m	Mean gradient of the channel
Drainage density	km/km^2^	Total stream length divided by the catchment area
Compactness	-	Gravelius compactness coefficient. Ratio between the catchment perimeter and the circumference of a circle with a surface equal to the catchment area

## 3. Results

### 3.1. National-scale assessment of stream network and river catchment characteristics

In this section we summarise selected characteristics across the Philippines, including: catchment area, trunk channel length, Gravelius compactness coefficient, drainage density, catchment relief, average catchment elevation, average catchment slope and average channel slope over 100 m segments. We present a consistent set of figures for each characteristic; raincloud plots [53] display the probability density and unclassed choropleth maps show their spatial distribution across catchments. To indicate deviations of stream network and river catchment characteristics from the national average, we normalised values by the mean average of the 128 catchments; normalisation provides a useful technique for contextualising the national-scale differences.

**Fig 3:**
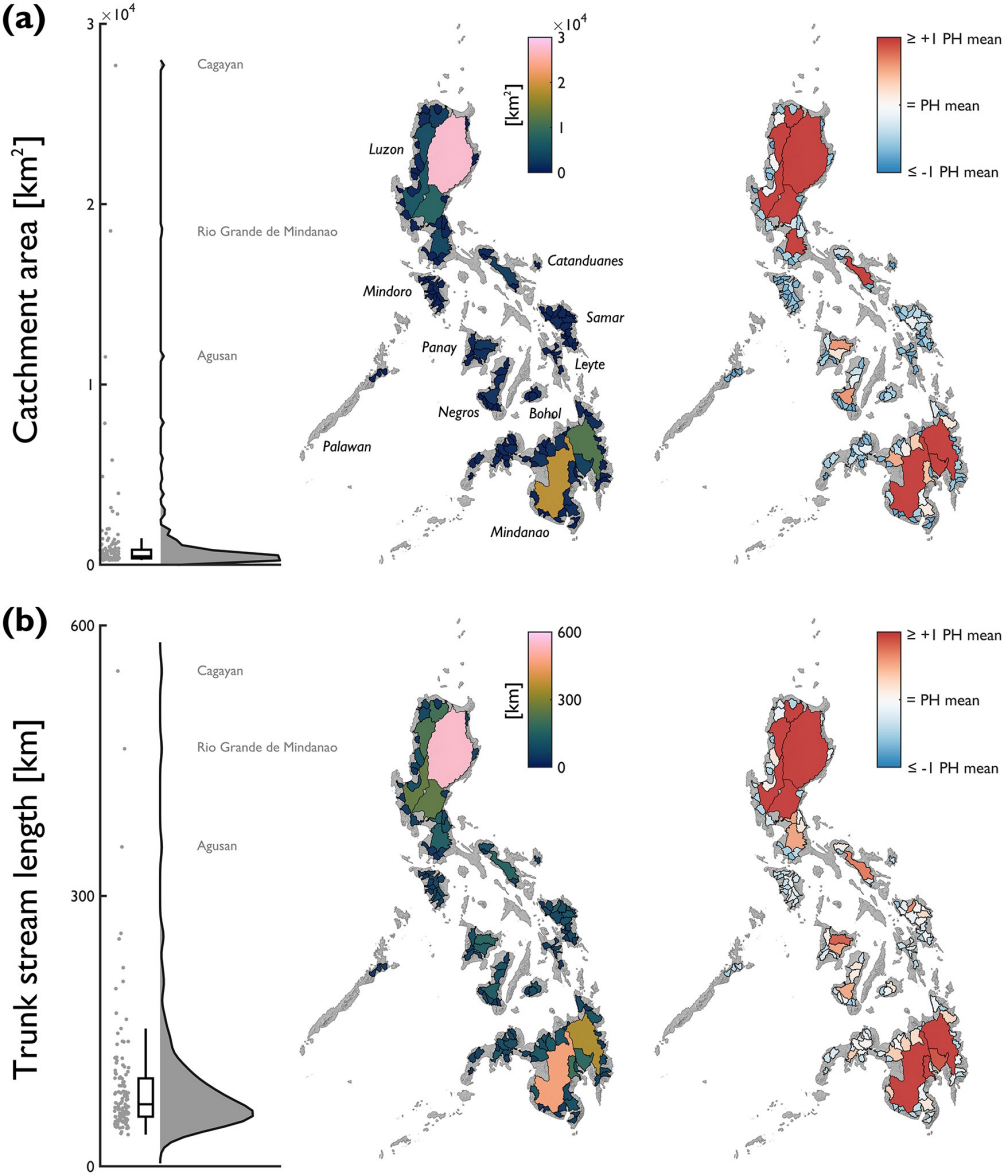
National-scale assessment of areal and linear characteristics expressed by: (a) catchment area; and, (b) trunk stream length. Note that for the red-blue choropleth map, values are displayed relative to the Philippine average for each attribute (catchment area = 1257 km^2^; trunk stream length = 90 km). Annotations on the raincloud plots denote catchments with notably high/low characteristics.

There are considerable differences in catchment area between the 128 catchments included in the analysis (Fig 3a). Catchment area ranges over two orders of magnitude from 258 to 27,648 km^2^ (median = 470 km^2^; mean = 1,257 km^2^; standard deviation = 3,154 km^2^). Three catchments have an area greater than 10,000 km^2^ (Cagayan 27,684 km^2^; Rio Grande de Mindanao 18,513 km^2^; Agusan 11,529 km^2^) and 25 catchments have an area greater than 1,000 km^2^. In general, the largest catchments are distributed on the islands of Luzon and Mindanao. Groups of smaller sized catchments with areas less than the national average tend to be distributed on smaller islands (e.g., Mindoro, Samar and Leyte).

An empirical power-law can be used to describe the relationship between the length of the longest channel (trunk stream) and catchment area [54]. It follows that the probability distribution for trunk stream length is similar in shape to the probability distribution for catchment area (Fig 3b). Trunk stream length varies over an order of magnitude from 35 to 549 km (median = 69 km; mean = 90 km; standard deviation = 71 km), with the longest trunk streams belonging to the largest catchments (Cagayan 549 km, Rio Grande de Mindanao 463 km; Agusan 354 km). Longest trunk streams tended to be located on the islands of Luzon and Mindanao.

**Fig 4:**
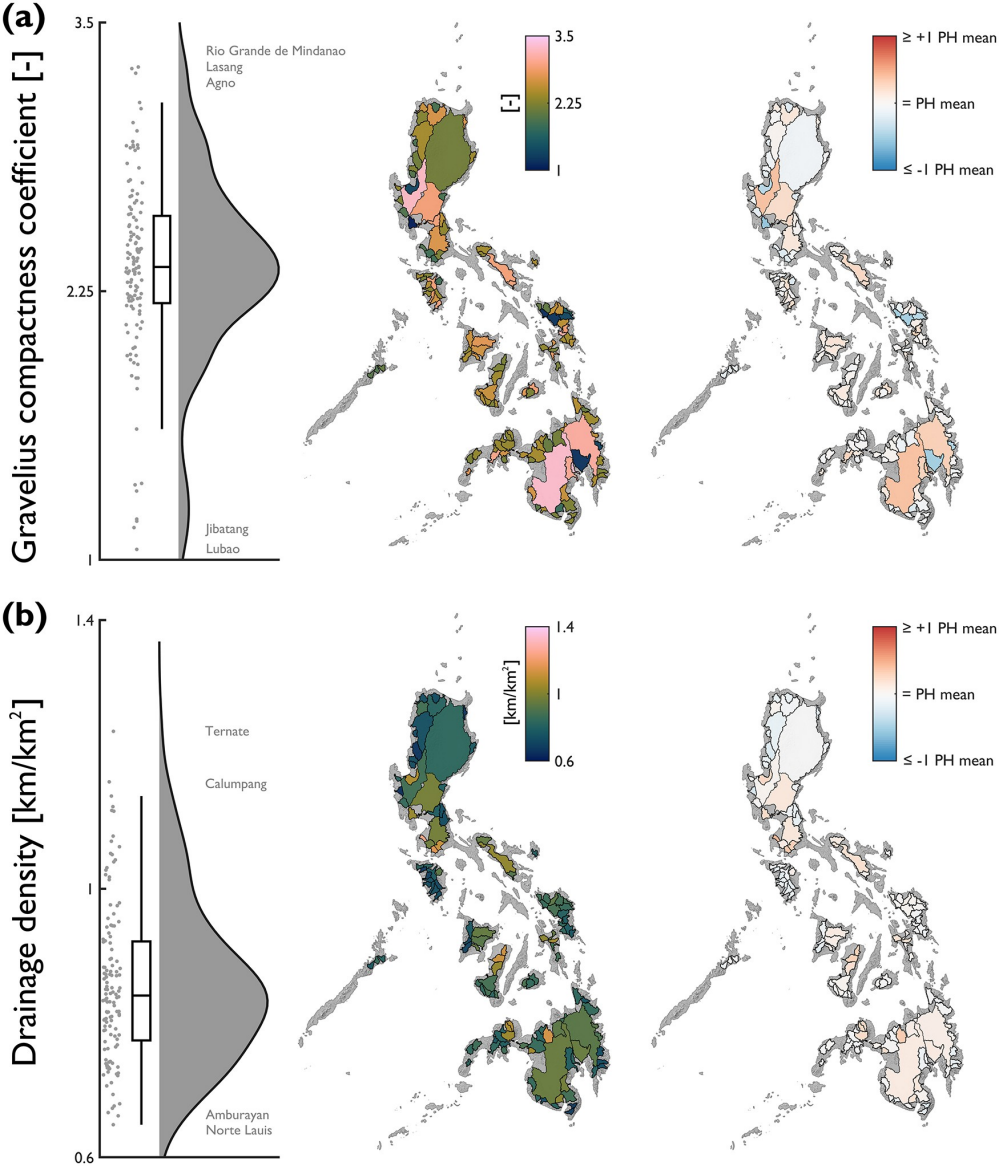
National-scale assessment of catchment shape and drainage characteristics expressed by: (a) Gravelius compactness coefficient; and, (b) drainage density. Note that for the red-blue choropleth map, values are displayed relative to the Philippine average for each attribute (Gravelius compactness coefficient = 2.36; drainage density = 0.86 km/km^2^). Annotations on the raincloud plots denote catchments with notably high/low characteristics.

Several morphometric characteristics exist to describe the shape or form of river catchments (e.g., circularity ratio [55]; elongation ratio [56]). Here we use the Gravelius compactness coefficient (GC) [57] for a size independent measure of catchment shape (Fig 4a). GC ranges from 1.05 to 3.29 (median = 2.36; mean = 2.36; standard deviation = 0.42); the range in values indicates a continuum of catchment shapes from almost circular to elongate and irregular. Catchment shape imparts a control on large-scale hydrological processes, influencing hydrograph responsiveness and the rate of peak flows [58]. Considerable differences in shape between neighbouring catchments (e.g., Tagum, GC = 1.18 and Agusan GC = 3.11) may contribute towards fundamental differences in their hydrological functioning given similarities in climate and tectonics.

Drainage density is another important landscape metric, providing a measure of landscape dissection, which exerts a control on flow and sediment transfer. It is spatially variable across the Philippines, but the magnitudes of absolute difference are small (Fig 4b). Drainage density ranges from 0.65 to 1.23 km/km^2^ (median = 0.84 km/km^2^; mean = 0.86 km/km^2^; standard deviation = 0.12 km/km^2^). Globally, patterns of drainage density are linked to dominant climatic zones, with wetter regions having higher drainage densities [59]. Given the marked spatial gradients in rainfall patterns across the Philippines [60] further analysis of the relationship with drainage density may be useful for future hydrological applications.

**Fig 5:**
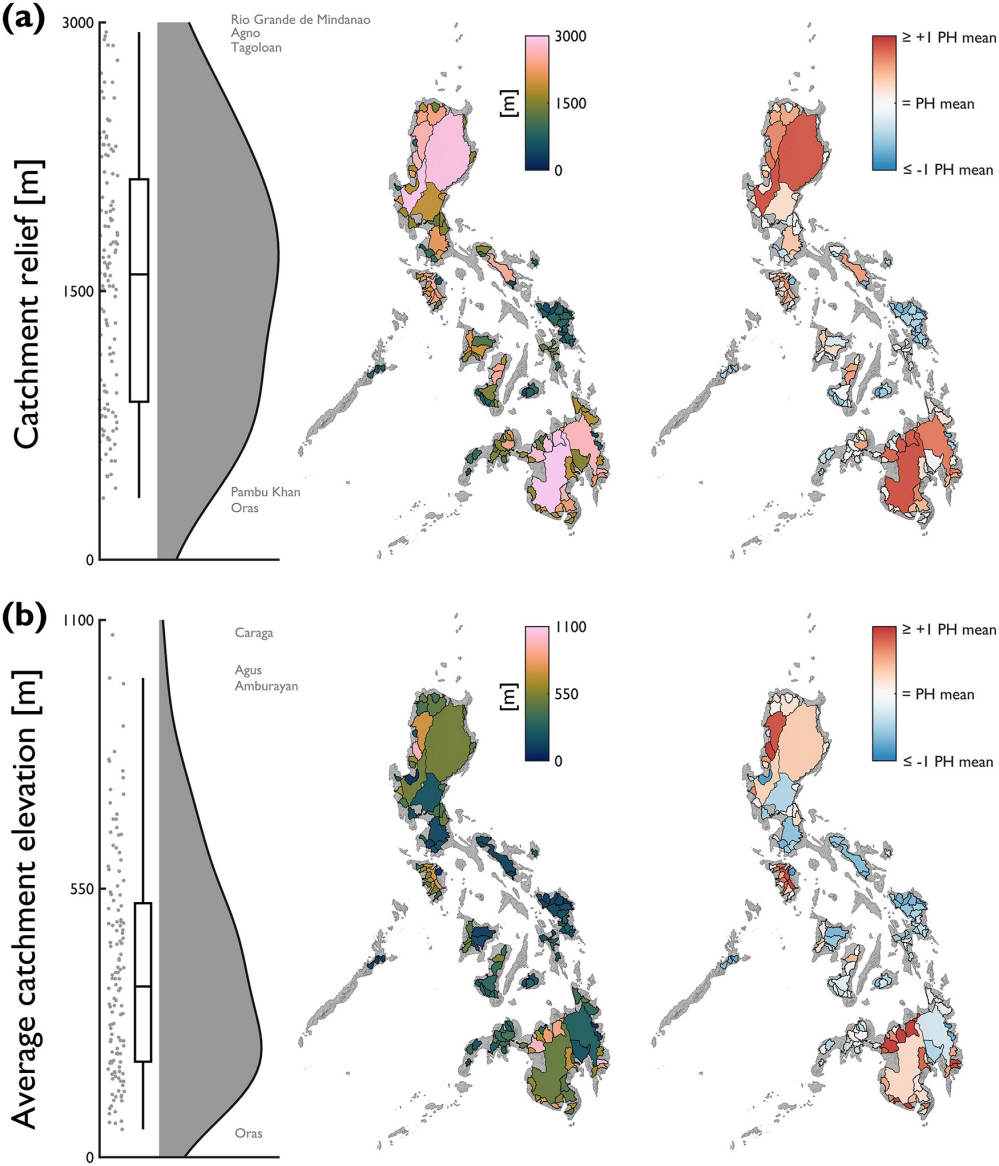
National-scale assessment of elevation characteristics, including: (a) catchment relief; and, (b) average catchment elevation. Note that for the red-blue choropleth map, values are displayed relative to the Philippine average for each attribute (catchment relief = 1563 m; average catchment elevation = 385 m). Annotations on the raincloud plots denote catchments with notably high/low characteristics.

Differences in elevation characteristics are considerable, as indicated by variation in catchment relief (Fig 5a) and average catchment elevation (Fig 5b). Relief ranges from 344 to 2,956 m (median = 1,593 m; mean = 1,563 m; standard deviation = 698 m). Catchments with highest relief have headwaters located in high elevation mountain ranges (e.g., Rio Grande de Mindanao = Kitanglad Mountain Range; Agno = Cordillera Central), whereas catchments with lowest relief tend to be distributed on low-lying islands (e.g., Oras = Samar). Mountainous headwaters are a common feature amongst the Philippine catchments; 34 catchments have relief greater than 2,000 m and 75 catchments have relief greater than 1,500 m. Average catchment elevation provides an additional measure of inter-catchment differences in elevation characteristics, ranging from 57 to 1,069 m (median = 350 m; mean = 385 m; standard deviation = 226 m). Clusters of catchments with average elevations greater than the national average are located in NW Luzon, Mindoro and Mindanao.

**Fig 6:**
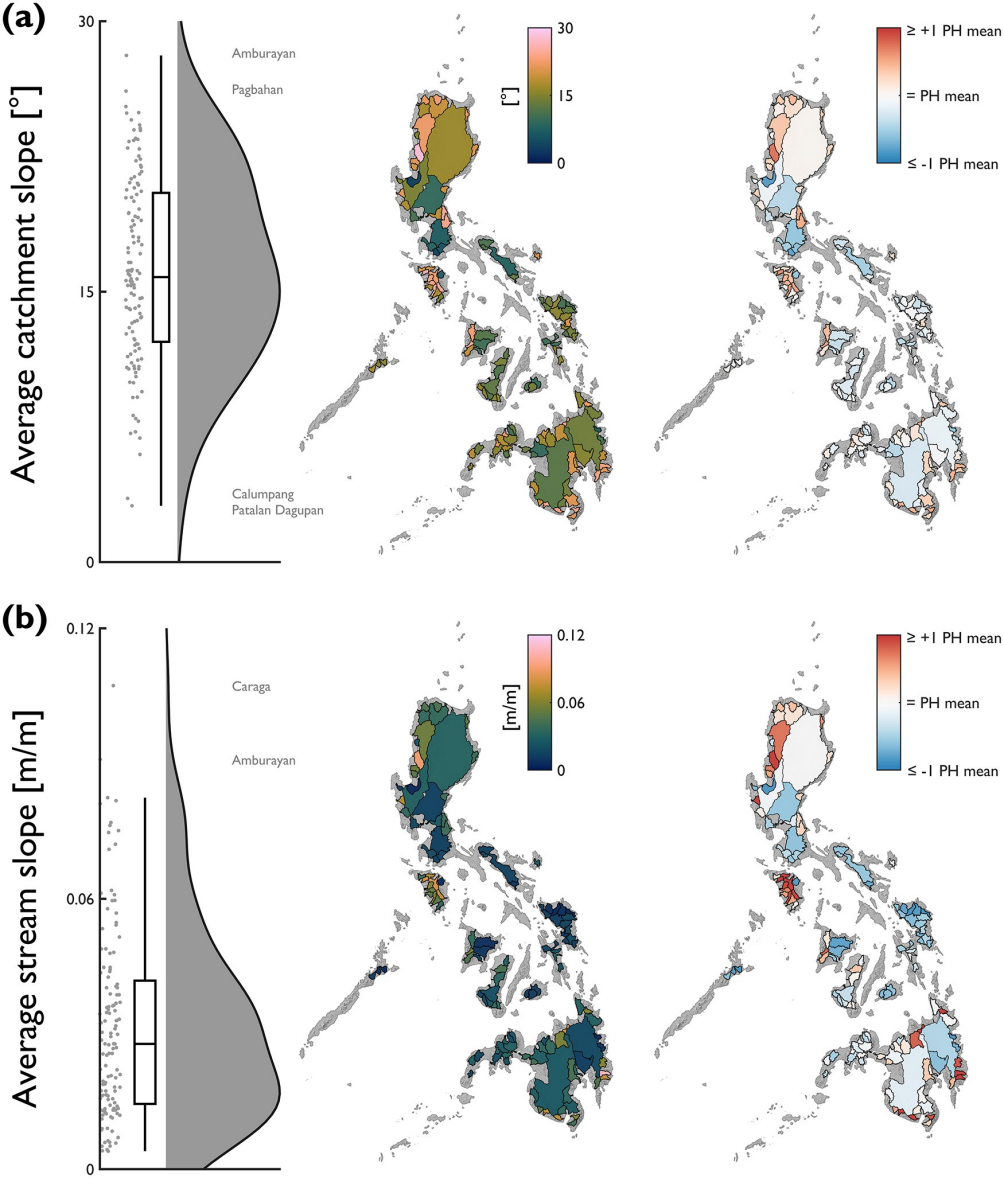
National-scale assessment of slope characteristics, including: (a) average catchment slope; and, (b) average stream slope. Note that for the red-blue choropleth map, values are displayed relative to the Philippine average (average catchment slope = 16.2°; average stream slope = 0.032 m/m). Annotations on the raincloud plots denote catchments with notably high/low characteristics.

Like elevation characteristics, there are substantial differences in slope characteristics between catchments (Fig 6). Average catchment slope ranges from 3.1 to 28.1° (median = 15.8°; mean = 16.2°; standard deviation = 5.2°). Average stream slope ranges by more than an order of magnitude from 0.004 to 0.107 m/m (median = 0.028 m/m; mean = 0.032 m/m; standard deviation = 0.021 m/m). Steeper catchments tend to be associated with steeper stream slopes (correlation coefficient = 0.76); a finding consistent with global relationships that have been developed over longer river segment lengths [61]. Catchments with steepest stream slopes tend to be smaller in size and located in the relatively high elevation clusters identified in NW Luzon, Mindoro and Mindanao.

### 3.2. Inter-catchment comparisons of topographic diversity: Do adjacent catchments differ?

In this section we make inter-catchment comparisons of topographic diversity. We appraise differences in elevation, catchment slope and channel slope between adjacent catchments. First we focus on three catchments in NW Luzon with headwaters in the Cordillera Central of Ilocos Norte (Bislak, Laoag and Abra). Then we focus on six catchments on Panay Island with headwaters in the Central Panay Mountain Range (Aklan, Cagaranan, Sibalom, Panay, Jalaur and Tigum). We comment on the spatial patterns of attributes across adjacent catchments and summarise their cumulative frequency distributions (CFD).

**Fig 7:**
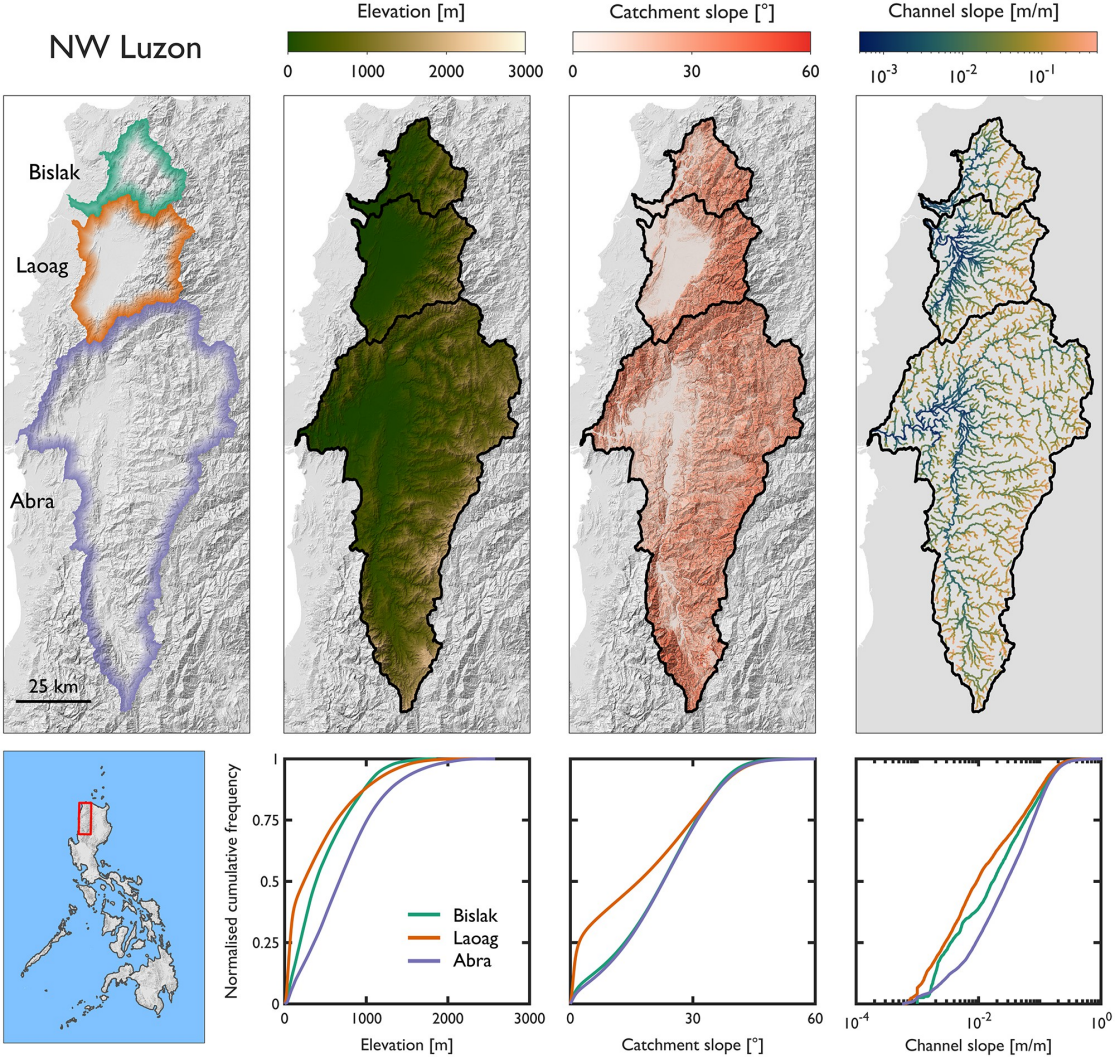
Inter-catchment comparisons of topographic diversity for three catchments in NW Luzon, the Philippines. All catchments have headwaters in the Cordillera Central of Ilocos Norte.

Our analyses reveal topographic similarities between adjacent catchments in NW Luzon (Fig 7 and Table 3). Although mean elevation and relief varies between the adjacent catchments, the CFD curves for elevation are similarly concave in shape; this indicates that the distribution of elevations are similar. In terms of catchment slope, the Bislak and Abra catchments are steepest (mean catchment slope = 21.9 and 22.4°). The large alluvial plain in the Laoag catchment results in a lower catchment-averaged slope value (mean catchment slope = 17.5°). The effect of the large alluvial plain is evident when comparing the 25^th^ percentiles of catchment slope (Bislak = 13.3°; Laoag = 2.1°; Abra = 13.8°); this is shown by the deviation from adjacent catchments in the CFD curve. Channels in the three catchments are generally steep; mean channel slope ranges from 0.037 m/m (Laoag) to 0.055 m/m (Abra). The slope of headwater channels are more consistent across all three catchments (90^th^ percentile of channel slope; Bislak = 0.131 m/m; Laoag = 0.116 m/m; Abra = 0.138 m/m). Although we observe some inter-catchment differences, catchment slope and channel slope in the NW Luzon catchments are steeper than the national-average (Fig 6). Results from NW Luzon demonstrate the potential for topographic similarities between adjacent catchments.

**Table 3:** Topographic attributes of the three catchments in NW Luzon.

Catchment	Area [km^2^]	Mean elevation [m]	Relief [m]	Catchment slope [°]						Channel slope averaged over 100 m segment [m/m]					
				Mean	10^th^	25^th^	50^th^	75^th^	90^th^	Mean	10^th^	25^th^	50^th^	75^th^	90^th^
Bislak	586	483	1860	21.9	4.0	13.3	22.7	30.8	36.9	0.044	0.002	0.004	0.017	0.058	0.131
Laoag	1262	404	2356	17.5	0.7	2.1	16.7	29.9	37.5	0.037	0.001	0.003	0.009	0.048	0.116
Abra	4893	726	2578	22.4	5.4	13.8	22.8	31.1	37.5	0.055	0.003	0.009	0.029	0.078	0.138

**Fig 8:**
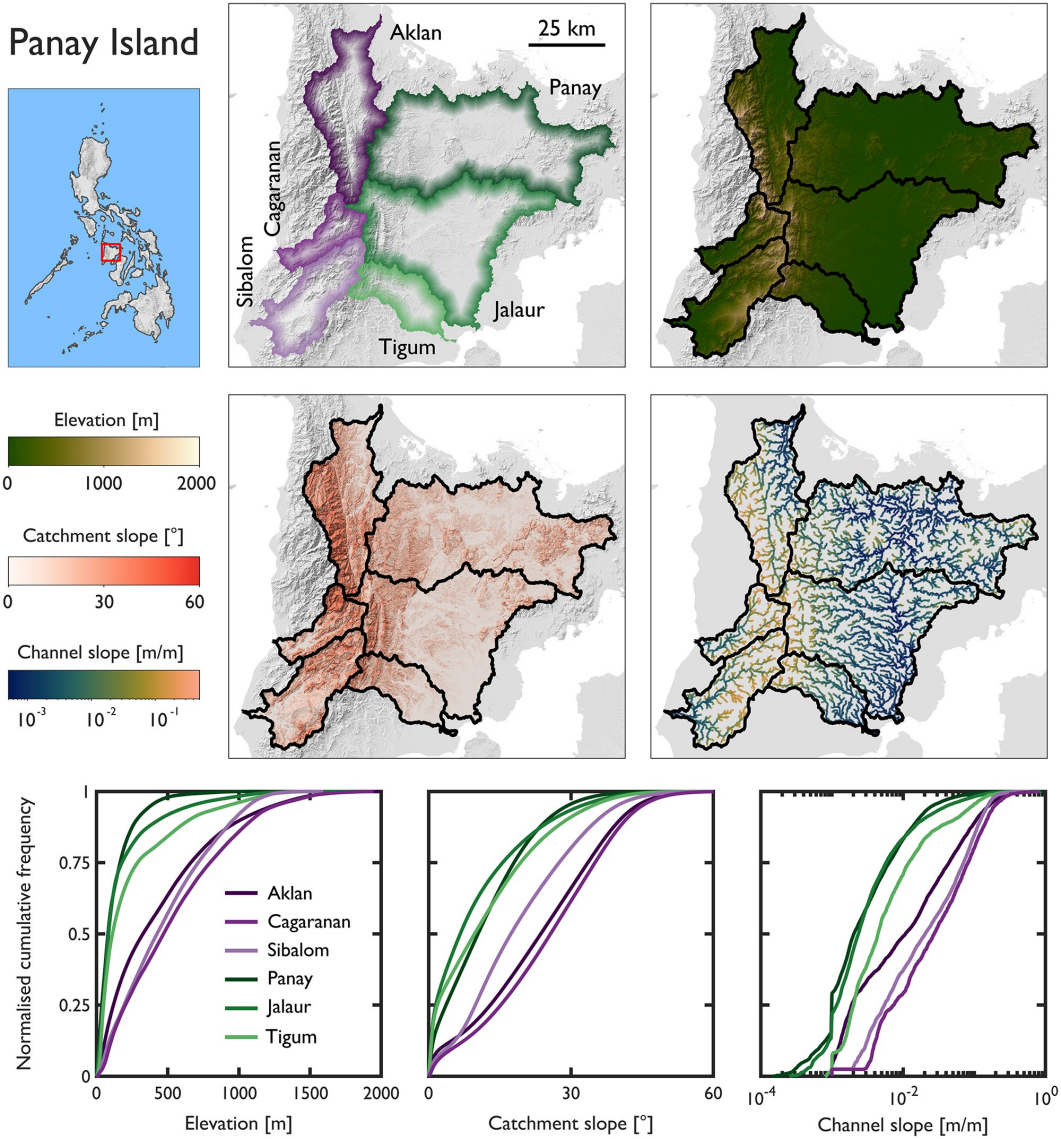
Inter-catchment comparisons of topographic diversity for six catchments on Panay Island, the Philippines. All catchments have headwaters in the Central Panay Mountain Range.

Topographic differences across the six adjacent catchments on Panay Island are substantial (Fig 8 and Table 4). Catchments draining to the west of the Central Panay Mountain Range (Aklan, Cagaranan, Sibalom) are characterised by markedly different topographic signatures than those catchments draining to the east (Panay, Jalaur and Tigum). Visually, the CFD curves for elevation, catchment slope and channel slope can be separated into those west and east draining catchments; the catchments draining to the west have greater elevations and are steeper. Averaging the summary statistics from Table 4, differences in mean catchment slope (west draining = 22.3°; east draining = 11.4°) and mean channel slope (west draining = 0.046 m/m; east draining = 0.012 m/m) are considerable. These differences are also apparent when comparing the slope of headwater channels (90^th^ percentile of channel slope; west draining = 0.126 m/m; east draining 0.029 m/m). The example from Panay Island demonstrates the potential for large inter-catchment differences in topography between adjacent catchments.

**Table 4:** Topographic attributes of the six catchments on Panay Island.

	Catchment	Area [km^2^]	Mean elevation [m]	Relief [m]	Catchment slope [°]						Channel slope averaged over 100 m segment [m/m]					
					Mean	10^th^	25^th^	50^th^	75^th^	90^th^	Mean	10^th^	25^th^	50^th^	75^th^	90^th^
*Draining west*	Aklan	891	436	2103	23.1	2.7	13.0	23.6	33.6	40.3	0.037	0.001	0.002	0.011	0.044	0.107
	Cagaranan	298	537	1946	24.9	5.2	15.2	25.7	35.1	41.5	0.057	0.004	0.007	0.027	0.081	0.149
	Sibalom	628	474	1592	19.0	4.0	9.8	17.3	27.5	35.6	0.045	0.003	0.005	0.020	0.071	0.123
*Draining east*	Panay	1996	124	1154	11.8	0.7	3.7	10.4	18.0	25.0	0.007	0.001	0.001	0.002	0.006	0.016
	Jalaur	1689	168	1940	10.2	0.4	1.5	6.7	15.6	26.0	0.010	0.001	0.001	0.002	0.006	0.019
	Tigum	412	237	1594	12.3	0.5	1.9	9.8	19.4	29.1	0.018	0.001	0.002	0.004	0.012	0.052

## 4. Discussion

### 4.1. Diversity and distinctiveness of stream networks and river catchments in the Philippines

Our national-scale assessment reveals the morphometric and topographic diversity of stream networks and river catchments in the Philippines. We observe marked variation in fundamental topographic characteristics and complex spatial patterns at the national-scale (Figs 3–6). Inter-catchment comparisons show the distinctive topographic signatures of individual catchments and the potential for topographic similarities and differences between adjacent catchments (Figs 7 and 8). Effective river management requires understanding of the resource that is being managed [62]. Our national-scale geodatabase provides an important step towards recognising the diversity and distinctiveness of stream networks and river catchments in the Philippines. The contrasts underline the importance of using place-based analyses for sustainable river management applications, respecting river diversity [63] and interpreting catchment-specific controls [64].

Tropical river catchments are known to be geomorphologically variable [65]; their stream networks express a variety of morphological forms [66] that contribute towards marked geomorphic diversity [67]. Shaped by gradients in elevation and their particular hydrological disturbance regime, tropical rivers are associated with substantial habitat heterogeneity and biological diversity [68]. At the catchment-scale, diversity in river morphology has been observed in the Philippines with eight distinct River Styles (geomorphic river types) identified in the 586 km^2^ Bislak catchment [40]. Spatial heterogeneity in the concavity index, used to indicate how quickly river channel gradient declines downstream, has been shown across the Cordillera Central of Ilocos Norte [69]. Physical attributes of the landscape impose boundary conditions that control hydromorphological attributes in river systems; the heterogeneity in morphometric and topographic characteristics are likely explained by the diverse geologic, tectonic and climatic settings across the analysed catchments.

### 4.2. Potential applications of the national-scale geodatabase

**Fig 9:**
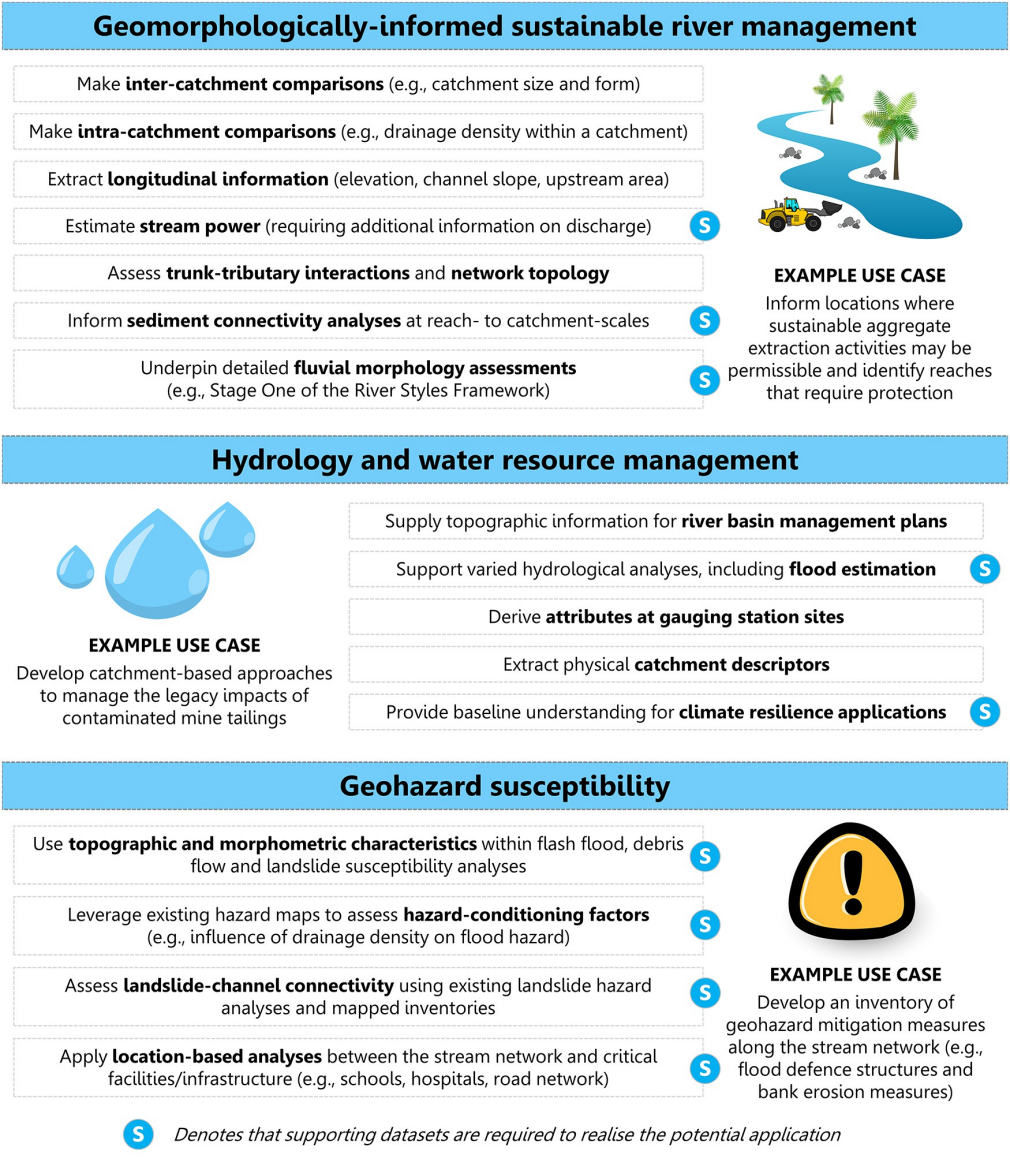
Potential applications of the national-scale geodatabase to river management applications in the Philippines.

Recognising the diversity of Philippine catchments, the national-scale geodatabase provides baseline data in support of varied river management applications across several thematic areas (Fig 9). When used independently or alongside supporting datasets, the national-scale geodatabase can contribute to potential applications that include:

- Geomorphologically-informed sustainable river management. The geodatabase provides a high-level overview of stream network and river catchment characteristics useful to a range of sustainable river management applications in the Philippines. The geodatabase allows users to make inter-catchment comparisons and contextualise local characteristics at the national-scale. For example, it enables relief characteristics to be compared across specific regions (e.g., how does the 90^th^ percentile of elevation vary across catchments on Luzon?). Datasets included in the geodatabase allow users to undertake bespoke intra-catchment analyses. For instance, the stream network can be used to investigate spatial variations in drainage density across individual catchments. The geodatabase provides longitudinal information that is fundamental to geomorphological applications (e.g., elevation, channel slope, upstream area). Longitudinal profiles are a key tool for analysing and visualising downstream patterns and controls in river systems [64]. The stream network allows imposed controls to be extracted (e.g., channel slope, upstream area) and flux controls to be estimated (e.g., stream power). Stream power is a widely used indicator of the capacity of rivers to erode and transport sediment [70, 71]. For the Bislak catchment in NW Luzon, spatially distributed patterns of total stream power were estimated when using the stream network and an area-discharge relationship derived for the region [40]; similar analyses can be upscaled and applied to many catchments in the Philippines where additional information on discharge is available. Moreover, the stream network can be used to assess drainage network configuration and tributary-trunk interactions, useful for interpreting landscape memory [72, 73]. Identification of river segments with similar topographic characteristics can inform sediment connectivity analyses at reach- to catchment-scales (e.g., when segmenting the stream network for geomorphic analyses [74–76]). Information from the geodatabase can underpin desk-based fluvial morphology assessments (e.g., application of Stage One of the River Styles Framework [64]), to be complemented by field-based observations. From a conservation and restoration perspective, topographic analyses are needed together with climatic, environmental and field-based insights to prioritise interventions and locate analogue reaches. Relevant to contemporary river management challenges in the Philippines, the geodatabase can be coupled with supplementary datasets and used as a resource base to inform sustainable aggregate extraction activities (e.g., identifying locations where sand and gravel mining activities should be restricted). Geomorphologically-informed knowledge can help when identifying locations where sediment connectivity and rates of replenishment are potentially high (and extraction activities may be permissible) and those locations where extraction activities would be unsustainable.
- Hydrology and water resources management. Systematic assessment of topographic characteristics will provide contextual information for river basin management plans. The geodatabase enables river basin managers to identify topographic similarities between geographically distinct catchments; this could offer new opportunities for collaboration, partnership and the sharing of best practice. Furthermore, the geodatabase can inform the subdivision of catchments based on similar topographic characteristics (e.g., slope); useful for developing targeted approaches to water resource management. The geodatabase will support varied hydrological analyses, including flood estimation. The stream network provides attribute information at gauging station sites; this information is particularly useful when locating and contextualising historical streamflow observations (e.g., [70, 71]). For ungauged catchments, elements of the United Kingdom Flood Estimation Handbook (FEH) can be drawn upon to extract physical catchment descriptors to be used as variables for flood prediction analysis [77]. Variables such as catchment area, drainage path length and mean catchment slope can be extracted and used to develop predictive equations. Looking to the future, projections from multiple dynamically downscaled climate model simulations suggest a tendency for wetter conditions to prevail over northern and central sections of the Philippines, particularly during the wet season [27, 78]. A baseline understanding of fundamental topographic characteristics will be important for predicting how hydrological regime change will manifest as part of climate resilience applications. Relevant to contemporary hydrology challenges in the Philippines, the geodatabase can support the development of catchment-based approaches to manage legacy impacts of mine-related contamination (e.g., [79]), geomorphologically-informed knowledge can contribute to improved understanding of the fate, transport and impact of contaminants. Using the geodatabase alongside additional information on climate characteristics (e.g., temperature and rainfall) would enhance catchment-based responses.
- Geohazard susceptibility. The geodatabase provides geospatial datasets commonly used as part of geohazard susceptibility analyses (e.g., for flash floods [80], debris flows [81] and landslides [82]). Used alongside existing hazard maps from NOAH, the geodatabase enables investigation of hazard-conditioning factors within catchments; an example would be to assess the contribution of stream network configuration to flood hazards. Relating to landslides, the geodatabase allows the quantitative assessment of landslide-channel connectivity when combined with existing datasets from mapped landslide inventories (e.g., [31]). Relevant to disaster risk reduction activities, location-based analyses can be undertaken to assess distances between the stream network and critical facilities or infrastructure (e.g., schools, hospitals, road network). Opportunities exist to develop detailed inventories of geohazard mitigation measures along the stream network (e.g., Environment Agency Spatial Flood Defences dataset [83]).

### 4.3. Limitations and future opportunities

The current study imposed a minimum catchment area threshold of 250 km^2^ when selecting catchments to include in the analyses; future work could apply the workflow to a large number of smaller-sized catchments (e.g., the 770 principal catchments outlined through the PHD Program). A limitation of the workflow was the treatment of large water bodies, which are represented as flat regions in the DEM. In the current workflow the stream network was hydrologically routed through the centre of flat regions, meaning that large water bodies were not excluded when calculating morphometric and topographic characteristics. For catchments with large lakes or reservoirs (e.g., Marikina Pasig, Pansipit and Agno) this introduces an unquantified source of error. Future work may seek to exclude these regions by masking out known water bodies prior to completing the topographic analyses; geospatial data from the Philippine Statistics Authority or OpenStreetMap could be used for this purpose. Moreover, the D8 algorithm used to derive flow direction may not perform well in low-relief topographic settings; more sophisticated flow direction algorithms could be used in future applications (e.g., MFD and Dinf).

By using the nationwide topographic data for this purpose, we have taken an important first step towards realising the potential of topographic data in the Philippines as part of geomorphological and hydrological applications. However, we acknowledge that there are limitations when delineating stream networks and river catchments from topographic data and are aware of data redundancy issues when using large numbers of morphometric and topographic characteristics. DEMs of varying spatial resolution and vertical accuracy cause differences when delineating stream networks that manifest as differences in morphometric and topographic characteristics [84]. To minimise these effects we used the highest quality topographic data available for the Philippines with nationwide coverage. Inter-catchment comparisons provide a useful starting point for interpreting topographically distinct catchments (e.g., separating high relief catchments from low relief catchments). However, a large numbers of morphometric and topographic characteristics are available and this can lead to considerable redundancy between derived characteristics [85]. We caution against drawing spurious conclusions and over-interpretations from morphometric and topographic characteristics, as has been known to occur in morphotectonic studies [86], and advocate for a data-interpretation-knowledge approach when analysing river systems [87].

Future geomorphological applications may seek to integrate local topographic analyses (e.g., confinement mapping [88]) and satellite imagery analyses (e.g., river channel change [89, 90]) into the national-scale geodatabase, to build more detailed understandings of river character and behaviour in the Philippines. Moreover, the workflow could be applied to other countries where high-quality topographic data are available, providing baseline products from systematic assessment of morphometric and topographic characteristics in support of varied river management challenges. Opportunities exist to link morphometric and topographic characteristics with additional geospatial datasets to generate more comprehensive geodatabases of hydro-environmental information. In support of catchment-scale hydrological applications, nationwide geodatabases containing hydro-environmental information have been developed in countries such as Brazil [91], Chile [92], the United Kingdom [93] and the United States [94]. At the global-scale, compendiums of descriptive hydro-environmental information are used to support of regional-scale hydro-ecological assessments [95]. New tools that can delineate river catchments, profile stream elevations and summarise geospatial datasets (e.g., RaBPRO [96]) will offer improvements in the data processing pipeline when producing hydro-environmental geodatabases in future applications.

## 5. Conclusions

This study provides a systematic assessment of fundamental topographic characteristics for 128 medium- to large-sized river catchments in the Philippines that will be used to underpin sustainable river management applications. Our national-scale assessment reveals the morphometric and topographic diversity of stream networks and river catchments. Variation between catchments is the key finding from our analyses. Catchments have a continuum of shapes (Gravelius compactness coefficient ranges from 1.05 to 3.29) and a range of drainage textures (drainage density from 0.65 to 1.23 km/km^2^). Average catchment slope ranges from 3.1 to 28.1° (mean = 16.2°) and average stream slope ranges by more than an order of magnitude from 0.004 to 0.107 m/m (mean = 0.032 m/m). Identifying divergences of morphometric and topographic characteristics from the national average provided an approach for identifying atypical regions (e.g., steeper than average stream networks in parts of NW Luzon and Mindoro; Fig 6b). Inter-catchment comparisons showed similarity in topographic signatures between adjacent catchments (Fig 7) but also marked topographic differences (Fig 8). By characterising and contextualising nationwide variations in hydromorphology we have demonstrated the topographic diversity and distinctiveness of river systems in the Philippines. We displayed our national-scale geodatabase of stream network and river catchment characteristics in an interactive ArcGIS web-application that enables users to freely access, explore and download the data. The geodatabase provides a baseline understanding of fundamental topographic characteristics in support of varied geomorphological, hydrological and water resource management and geohazard susceptibility applications.
